# Effectiveness and safety of warm needling therapy combined with electroacupuncture for patients with plantar heel pain syndrome: A protocol for systematic review and meta-analysis

**DOI:** 10.1097/MD.0000000000029171

**Published:** 2022-05-27

**Authors:** Juecan Wu, Jinghua Lu, Chengyang Jiang

**Affiliations:** a Department of Acupuncture and Tuina, Hangzhou Cancer Hospital, Hangzhou, Zhejiang, China; b Health Clinic, Zhejiang Police Officer Vocational College, Dongyang, Zhejiang, China; c Department of Acupuncture and Tuina, Hangzhou Traditional Chinese Medicine Hospital, Hangzhou, Zhejiang, China.

**Keywords:** electroacupuncture, meta-analysis, plantar heel pain syndrome, protocol, warm needling

## Abstract

**Background:**

Several studies have reported positive therapeutic effects of electroacupuncture, warm needling, or a combination of the 2 for heel pain; however, the quality of the evidence is limited by methodological limitations. Given that there are no high-quality meta-analyses or reviews incorporating the available evidence, the aim of this study was to systematically review the level I evidence in the literature to determine whether a combination of electroacupuncture and warm needling therapy is more beneficial than acupuncture alone in patients with plantar heel pain syndrome.

**Methods:**

From the inception to May 2022, the Wanfang, CNKI, EMBASE, PubMed, Web of Science, and Cochrane Library electronic databases will be searched using the key phrases “acupuncture”, “warm needling”, “electroacupuncture”, “heel pain”, “plantar pain”, and “prospective” for all relevant studies. The outcomes include pain, physical disability, plantar fascia thickness, and foot functional status. Quality assessment of all studies included in this review will be independently assessed by 2 reviewers using the Cochrane Collaborations tool. We consider significant heterogeneity between trials if *I*^2^ > 50%, and severe heterogeneity if *I*^2^ > 75%. When significant heterogeneity is indicated, we will find the source of heterogeneity by subgroup or sensitivity analysis.

**Results:**

The results of our review will be reported strictly following the Preferred Reporting Items for Systematic Reviews and Meta-Analyses reporting guidelines and the recommendations of the Cochrane Collaboration.

**Conclusions:**

We initially hypothesized that combination therapy would lead to better treatment outcomes.

Registration number: 10.17605/OSF.IO/VWBYJ.

## 1 Introduction

Heel pain is a significant cause of disability, accounting for 15% of clinician referrals for foot pain management.^[1]^ Plantar heel pain syndrome (PHPS) is defined as acentralised plantar heel pain with soreness and tenderness of the heel. Pain may radiate from the central heel pad or medial calcaneal tubercle and may extend along the plantar fascia to the medial longitudinal arch of the foot.^[2]^ The severity of pain can range from irritating to disabling. It is usually more pronounced on standing up after rest and with extension (dorsiflexion) of the foot and big toe. The peak incidence occurs between the ages of 50 and 60, and it is more common in women.^[3]^

Conventional treatment options for PHPS used in clinical practice include device therapy, physical therapy, medication, and surgery. However, there is no consensus on the optimal treatment for PHPS. Acupuncture is a traditional Chinese medical technique that has been used to treat a variety of musculoskeletal pain-related conditions. Currently, clinicians can use a variety of acupuncture modalities, such as electroacupuncture, warm acupuncture, and hand acupuncture.^[4,5]^ Stimulation of acupuncture points by acupuncture has been shown to induce analgesia by releasing neuropeptides such as enkephalins, dynorphins, beta-endorphins, and endorphins.^[6]^ A recent systematic review suggests that acupuncture can reduce PHPS pain in the short term, however, more nuanced issues, such as how practitioners choose different acupuncture in different situations, remain unclear.^[7]^

Previous studies have reported electroacupuncture for analgesia.^[8,9]^ Warm needling therapy is an indirect moxibustion method that can be combined with acupuncture. It uses ignited moxa sticks to apply heat to certain points or areas on the body surface to treat diseases. During warm needling, the heat generated by the burning of the moxa sticks passes through the needles. The body is transmitted to the corresponding acupuncture points by means of radiation and conduction, thereby stimulating the deep tissue within the acupoints and warming the surface acupoints at the same time.^[10]^ Several studies have reported positive therapeutic effects of electroacupuncture, warm needling, or a combination of the 2 for heel pain; however, the quality of the evidence is limited by methodological limitations.^[11,12]^ Combining these 2 modalities may provide a beneficial synergy in the treatment of PHPS. Given that there are no high-quality meta-analyses or reviews incorporating the available evidence, the aim of this study was to systematically review the level I evidence in the literature to determine whether a combination of electroacupuncture and warm needling therapy is more beneficial than acupuncture alone in patients with PHPS.

## 2 Materials and methods

### 2.1 Search strategy and registration

Two independent investigators will follow the Preferred Reporting Items for Systematic Reviews and Meta-Analyses (PRISMA) reporting guidelines and the recommendations of the Cochrane Collaboration to conduct the present meta-analysis. From the inception to May 2022, the Wanfang, CNKI, EMBASE, PubMed, Web of Science, and Cochrane Library electronic databases will be searched using the key phrases “acupuncture”, “warm needling”, “electroacupuncture”, “heel pain”, “plantar pain”, and “prospective” for all relevant studies. Moreover, references cited by the relevant sources will be also hand-searched to identify any additional articles that can not be found in our database query. There are no language restrictions. Ethical approval is not necessary because the present meta-analysis will be performed on the basis of previous published studies. The systematic review protocol has been registered on Open Science Framework registries. The registration number is 10.17605/OSF.IO/VWBYJ. We will update our protocol for any changes in the entire research process if needed.

### 2.2 Inclusion and exclusion criteria

#### 2.2.1 Types of study

Randomized controlled trial.

#### 2.2.2 Intervention

Combined therapies including electroacupuncture and warm needling.

#### 2.2.3 Control

A single acupuncture treatment, such as electro-acupuncture or warm needling.

#### 2.2.4 Patients

Participants with PHPS.

#### 2.2.5 Outcomes

Pain, physical disability, plantar fascia thickness, and foot functional status.

Exclusion criteria include the following items: non-randomized controlled trials or duplicate publications; animal studies; case reports and reviews; conference abstracts; no clinical studies comparing combination therapy and single acupuncture or moxibustion; data in text could not be extracted.

### 2.3 Study selection and data extraction

First, 2 reviewers will independently screen the retrieved literature and use Endnote to manage the retrieved literature to remove duplicates. Then, an initial screening by reading titles and abstracts exclude irrelevant studies, especially studies on animal experiments, expert experience, incomplete articles, review articles, and case reports. Further screening will be performed by reading the full text to remove studies showing intervention mismatches, missing data, and non-randomized controlled trials. In addition, any disagreements will be resolved by discussion with the third reviewer. After screening, relevant information will be extracted from the final included studies, including authors, year of publication, interventions, sample information, treatment course, key elements of risk of bias assessment, outcome measures, and outcome measures, etc.

### 2.4 Statistical analysis

In the review, we will perform a meta-analysis using Review Manager software (RevMan v. 5.3). To assess the effect of combined acupuncture treatment on heel pain, hazard ratios and 95% confidence intervals will be used to analyze dichotomous data, and mean differences and 95% confidence intervals or standardized mean differences and 95% confidence intervals will be used to analyze continuous data. Chi-square and *I*^2^ tests will be used to assess statistical heterogeneity. We consider significant heterogeneity between trials if *I*^2^ > 50%, and severe heterogeneity if *I*^2^ > 75%. When significant heterogeneity is indicated, we will find the source of heterogeneity by subgroup or sensitivity analysis. Subgroup analyses will be performed according to treatment period, and each heterogenous trial is excluded for sensitivity analyses. If there is substantial heterogeneity, a random-effects model will be used; otherwise, a fixed-effects model will be used to synthesize the data. Descriptive synthesis of study results will be performed if the number of appropriate studies was only 1, or if the data are not suitable for quantitative synthesis. Funnel plots will be used to assess publication bias if the number of pooled studies exceeded 10.

### 2.5 Risk of bias and quality assessments

Quality assessment of all studies included in this review will be independently assessed by 2 reviewers using the Cochrane Collaborations tool. Seven criteria are applied: random sequence generation, allocation concealment, blinding of participants and personnel, blinding of outcome assessment, incomplete outcome data, selective reporting, and other biases (defined as comparability of baseline data). For each item, the assessment is classified as low risk, high risk, or unclear risk according to the description of the methods in each study. Any disagreements will be resolved by discussion with the third author.

## 3 Discussion

Plantar heel pain is common. It may result from different conditions, such as plantar fasciitis, tarsal tunnel syndrome, entrapment of the plantar nerves in the foot, calcaneal fracture, rupture of the plantar fascia, and atrophy of the heel fat pad. Several studies have reported positive therapeutic effects of electroacupuncture, warm needling, or a combination of the 2 for heel pain; however, the quality of the evidence is limited by methodological limitations.^[11,12]^ Combining these 2 modalities may provide a beneficial synergy in the treatment of PHPS. Given that there are no high-quality meta-analyses or reviews incorporating the available evidence, the aim of this study was to systematically review the level I evidence in the literature to determine whether a combination of electroacupuncture and warm needling therapy is more beneficial than acupuncture alone in patients with PHPS.

## Author contributions

**Conceptualization:** Juecan Wu.

**Data acquisition:** Juecan Wu, Chengyang Jiang, Jinghua Lu.

**Data analysis:** Juecan Wu, Chengyang Jiang, Jinghua Lu.

**Data curation:** Jinghua Lu, Juecan Wu.

**Definition of intellectual content:** Juecan Wu.

**Formal analysis:** Jinghua Lu, Juecan Wu.

**Funding acquisition:** Chengyang Jiang.

**Investigation:** Jinghua Lu, Juecan Wu.

**Literature research:** Juecan Wu.

**Manuscript editing:** Juecan Wu.

**Manuscript preparation:** Juecan Wu.

**Manuscript review:** Juecan Wu.

**Methodology:** Juecan Wu.

**Project administration:** Chengyang Jiang.

**Resources:** Chengyang Jiang.

**Statistical analysis:** Juecan Wu, Chengyang Jiang.

**Study concepts:** Juecan Wu.

**Study design:** Juecan Wu.

**Supervision:** Chengyang Jiang.

**Validation:** Jinghua Lu.

**Writing – original draft:** Juecan Wu.

**Writing – review & editing:** Chengyang Jiang, Juecan Wu.
